# Characterization of Low-Cost Capacitive Soil Moisture Sensors for IoT Networks

**DOI:** 10.3390/s20123585

**Published:** 2020-06-25

**Authors:** Pisana Placidi, Laura Gasperini, Alessandro Grassi, Manuela Cecconi, Andrea Scorzoni

**Affiliations:** Dipartimento di Ingegneria, University of Perugia, via G. Duranti, 93, 06125 Perugia, Italy; laura.gasperini2@studenti.unipg.it (L.G.); alessandro.grassi@studenti.unipg.it (A.G.); manuela.cecconi@unipg.it (M.C.); andrea.scorzoni@unipg.it (A.S.)

**Keywords:** moisture sensor, capacitive measurement, capacitive moisture sensor, soil water content measurement techniques, geotechnical investigation, SKU:SEN0193 sensor

## Abstract

The rapid development and wide application of the IoT (Internet of Things) has pushed toward the improvement of current practices in greenhouse technology and agriculture in general, through automation and informatization. The experimental and accurate determination of soil moisture is a matter of great importance in different scientific fields, such as agronomy, soil physics, geology, hydraulics, and soil mechanics. This paper focuses on the experimental characterization of a commercial low-cost “capacitive” coplanar soil moisture sensor that can be housed in distributed nodes for IoT applications. It is shown that at least for a well-defined type of soil with a constant solid matter to volume ratio, this type of capacitive sensor yields a reliable relationship between output voltage and gravimetric water content.

## 1. Introduction

The development of the Internet of Things (IoT) refers to a global network of intelligent objects, or “things,” based on sensors, i.e., microcontrollers augmented with networking capabilities. In this framework, communication technologies can improve the current methods of monitoring, supporting the response appropriately in real time for a wide range of applications [1,2,3,4]. Sensors are designed for collecting information (e.g., temperature, pressure, light, humidity, soil moisture, etc.) whereas network-capable microcontrollers are able to process, store, and interpret information, building intelligent wireless sensor networks (WSN) [5,6,7].

WSNs have extensively been adopted in agriculture since their first introduction in the new century [8,9]. A distinct advantage of wireless transmission is a significant reduction and simplification in wiring and harness, a required feature for smart farming [10,11], where installation flexibility for sensors is not an option. A description of a modular IoT architecture for several applications including but not limited to healthcare, health monitoring, and precision agriculture is reported in previous works [12,13].

In this scenario, soil moisture or soil water content is a matter of great importance. In fact, water is considered as one of the most critical resources for sustainable development because in forthcoming years fresh water will increasingly be used in irrigated areas, in towns for domestic purposes, and in the industry. Furthermore, the efficiency of irrigation is very low. It is well known that only a fraction of the irrigation water is actually used by the crops. Hence, the sustainable use of irrigation water is a main concern for agriculture and not only under scarcity conditions. Considerable efforts have been allocated over time to increase water efficiency based on the assertion that “more can be achieved with less water through better management” [14].

Modernization and automated scheduling of irrigation systems demands for sensor-based equipment. Traditionally, sensor data are associated with environmental conditions and soil water status to provide information about the full crop water requirements [15]. The most commonly used soil parameter sensors exploit dielectric properties, since they are relatively cheap and flexible [16,17]. Their correct operation requires complex calibration, taking into account aspects such as soil texture and structure, temperature, and water salinity [10,17,18,19], as well as the spatial variability of soil conditions [20]. Thermal and multispectral cameras, satellites, or infrared radiometers (IR) are also used to estimate water crop needs [21,22,23,24].

Nonetheless, the experimental and accurate determination of soil moisture is also a matter of great importance in different scientific fields, such as agronomy, soil physics, geology, hydraulics, and soil mechanics. Physical, chemical, mineralogical, and biological properties are also affected by the soil water content. A summary of the state of the art soil moisture measurement techniques has been previously reported [24,25,26,27,28]. The thermo-gravimetric technique is the classical soil moisture (or water content) measurement method for geotechnical engineering applications. Modern techniques include soil resistivity detection, neutron scattering, tensiometers, infrared moisture balance, dielectric techniques like frequency domain reflectometry, time domain reflectometry, heat flux soil moisture sensors, optical techniques, and modern micro-electromechanical systems. In particular, several reviews have been published in the literature on dielectric methods [29,30,31].

Our paper focuses on the experimental characterization of a commercial, low-cost “capacitive” soil moisture sensor that can be housed in distributed nodes for IoT applications with the aim to validate its performance and soundness in the determination of soil physical properties. IoT sensor nodes should be deployed in large numbers and the cost of the components of the nodes should be minimized. The chosen sensor, identified as SKU:SEN0193, is the cheapest and most easily available in the market. However, detailed information on the sensor operation is not available. Therefore, it is useful to investigate its performance and understand its limits both for irrigation management and for soil moisture determination, e.g., in geotechnical applications. To the best of our knowledge, the sensor studied in the present paper has been characterized only once before [32], when it as evaluated for accuracy and reliability under laboratory conditions. The authors found that this sensor did not perform acceptably in predicting soil moisture content in a laboratory soil mixture prepared by mixing organic-rich soil and vermiculite, while it can estimate soil water in gardening soil in the so-called “field capacity” range. The present paper adds an in-depth characterization of the electrical circuit of the sensor and a statistical analysis on a number of nominally identical samples with the aim to better understand its limits and applicability with silica sandy soil.

## 2. Soil Water Content Measurements

Undoubtedly, water plays an essential role in the chemical-physical-mechanical properties of soil. With regard to the upper soil volumes close to the ground table, plant growth, organization of natural ecosystems, and biodiversity, are surely affected by soil moisture quantity and its variations. In the agriculture sector, application of adequate and timely irrigation, depending upon the soil-moisture-plant environment, is essential for crop production. More generally, soil vegetation is certainly affected by soil moisture, among others. Therefore, quantitative evaluation of soil water content from the ground surface to larger depths is a key-issue for the comprehension and assessment of many phenomena depending on the interaction soil-vegetation-atmosphere, such as soil erosion, runoff, and soil water infiltration. The subject is complex since the study of these processes requires specific skills in the field of soil physics, agronomy, hydraulics, and soil mechanics. Soil vegetation modifies the hydrological balance of the involved area due to both the aerial plant *apparati* to capture part of the water rainfall and the capacity of the plants to adsorb water from the surrounding soil and transfer it to the atmosphere through evapotranspiration. The latter mechanism may yield a reduction of the soil degree of saturation (an increase of suction) and consequently an increase of the soil shear strength. Thus, soil vegetation may also have an important effect in the engineering field of slope stability and environmental protection [33,34].

Hence, the determination of soil water content, degree of saturation, and their variations upon environmental conditions is crucial in the different fields dealing with the soil behavior. Pore pressures pertaining to the soil fluid phase (gas + water) also play a fundamental role in the mechanical behavior of soils. In turn, the latter impacts the performance of geotechnical structures and systems, such as foundations, earth retaining walls, slopes, and so on. 

**Figure 1:**
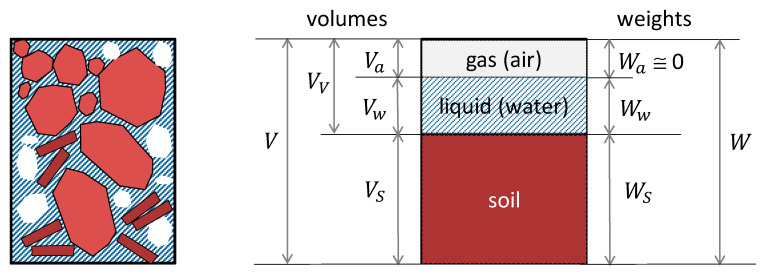
Soil-phase relationships.

Looking at the scale of a soil element, by being a porous material, this is intrinsically multiphase. Typically, three distinct phases are recognized: solid (mineral particles), liquid (usually water), and gas. In the framework of soil mechanics, the relationships among the soil phases are schematically represented in Figure 1, which facilitates the definition of the phase relationships. Although the quantities plotted in the figure and their relationships are very well established in the fields of soil mechanics, for the sake of completeness it is only the case to recall the definitions of the main quantities addressed in this paper. With regard to volume-relationships, porosity, voids ratio, degree of saturation, and volumetric water content, they are defined as follows. By considering a soil element, porosity (*n*) is the ratio of voids (pores) volume to total volume, while the voids ratio (*e*) is the ratio of voids volume to solid volume. The degree of saturation (S_*r*_) is defined as the percentage of the voids volume filled with water (S_*r*_ = 0 for a dry soil; S_*r*_ = 1 for a saturated soil; S_*r*_ < 1 for a partially saturated soil). On the other hand, the most useful relationship between phases in terms of weights is the gravimetric water content of a soil element: in geotechnics and soil science it is defined as the weight of water divided by the weight of solid.

Based on weight measures, the gravimetric water content is readily obtained in a laboratory environment:

(1) $$w=\frac{W_{w}}{W_{s}}=\frac{W-W_{s}}{W_{s}},$$

This is accomplished by weighing the natural soil (w), drying it in an oven, then weighing the dry soil, measuring the weight $w_{s}$, and computing the water content according to Equation (1). Let us define $\gamma_{dry}$ as the dry unit weight and $\gamma_{w}$ as the unit weight of water (≅10 kN/m^3^):

(2) $$\gamma_{dry}=\frac{W_{s}}{V}\;\gamma_{w}=\frac{W_{w}}{V_{w}},$$

Finally, the volumetric water content (*θ_w_*) is the ratio of the water volume to the total volume:

(3) $$\theta_{w}=\frac{V_{w}}{V}=w\cdot \frac{\gamma_{dry}}{\gamma_{w}}$$

We underline in Equation (3) the gas volume of the soil is included in the dry unit weight $\gamma_{dry}$.

## 3. Capacitive Moisture Sensor

**Figure 2:**
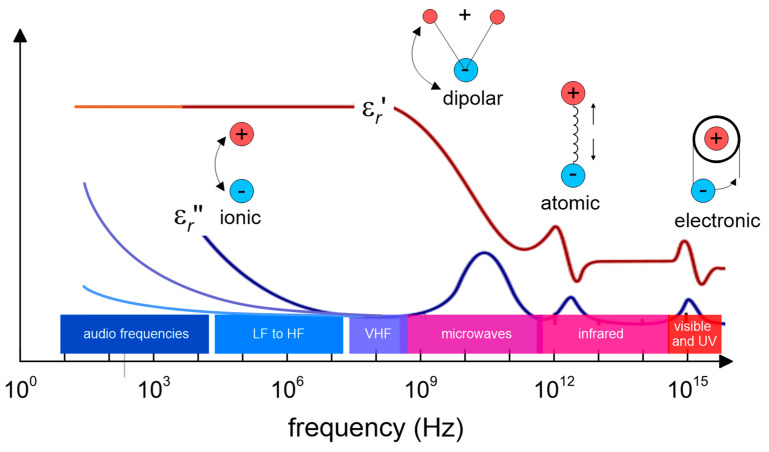
Dielectric mechanisms contributing to dielectric behavior at the microscopic level. Molecular relaxation (dipolar rotational, atomic vibrational and electronic energy states) have been highlighted. With respect to a similar picture in [36], different branches of $\varepsilon_{r}^{\prime \prime }$ are drawn, corresponding to different values of soil electrical conductivity $\sigma_{dc}$. Electromagnetic wave ranges have also been emphasized.

In this paper, a capacitance probe has been used for sensing moisture. It is well known [35] that the output of a capacitive moisture sensors depends on the complex relative permittivity $\varepsilon_{r}^{*}$ of the soil (dielectric medium):

(4) $$\varepsilon_{r}^{*}=\varepsilon_{r}^{\prime }-j\varepsilon_{r}^{\prime \prime }=\varepsilon_{r}^{\prime }-j\left(\varepsilon_{relax}^{\prime \prime }+\frac{\sigma_{dc}}{2\pi f\varepsilon_{0}}\right),$$

where $\varepsilon_{r}^{\prime }$ and $\varepsilon_{r}^{\prime \prime }$ are the real and the imaginary part of the permittivity, respectively (Figure 2), $\sigma_{dc}$ is the electrical conductivity, $\varepsilon_{relax}^{\prime \prime }$, the molecular relaxation contribution (dipolar rotational, atomic vibrational, and electronic energy states), *j* is the imaginary number $\sqrt{-1}$, and *f* is the frequency. The real part of permittivity ($\varepsilon_{r}^{\prime }$) quantifies the amount of energy from an external electric field being stored in a material. The imaginary part of permittivity ($\varepsilon_{r}^{\prime \prime }$), also dubbed the “loss factor”, measures how dissipative or lossy a material is to an external electric field: $\varepsilon_{r}^{\prime \prime }$ > 0. Losses are associated with two main processes: molecular relaxation and electrical conductivity. Permittivity is dependent on (i) frequency, (ii) moisture, and the (iii) salinity and ionic content of the soil.

A number of lossy dielectric mechanisms contribute to the global permittivity, being that the ionic and the rotational dipolar effects the most significant ones (see Figure 2). For increasing frequency only, the fast mechanisms survive. Every cutoff frequency is characterized by a sudden decrease of $\varepsilon_{r}^{\prime }$ and a peak of $\varepsilon_{r}^{\prime \prime }$.

The electrical equivalent circuit of a capacitive sensor always includes an element whose capacitance can be written as:

(5) $$C=\varepsilon_{r}^{*}\varepsilon_{0}G_{0}$$

where $G_{0}$ is a geometric factor and $\varepsilon_{0}$ is the permittivity in a vacuum. 

The dielectric is able to store energy when an external electric field is applied. If an AC sinusoidal voltage source v of frequency *f* is placed across the capacitor (no relaxation medium) a charging current *i_c_* and a loss current *i_l_* that are related to the dielectric constant will be made up.

(6) $$i=j\omega Cv=\left(j\omega \varepsilon_{r}^{\prime }+\omega \varepsilon_{r}^{\prime \prime }\right)\varepsilon_{0}G_{0}v$$

where ω is the angular velocity *ω* = 2π*f* and *v* and *i* are phasors.

**Figure 3:**
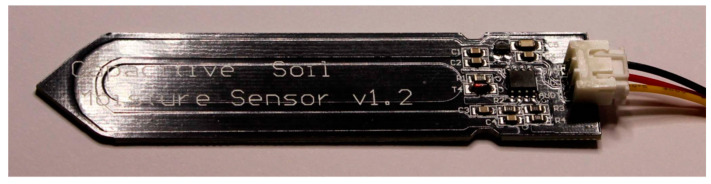
A “capacitive” soil moisture sensor. The grazing light image shows the coplanar concentric capacitor of the sensor.

In Figure 3 the used commercial, blade-shaped, “Capacitive Soil Moisture Sensor v1.2” is portrayed. 

**Figure 4:**
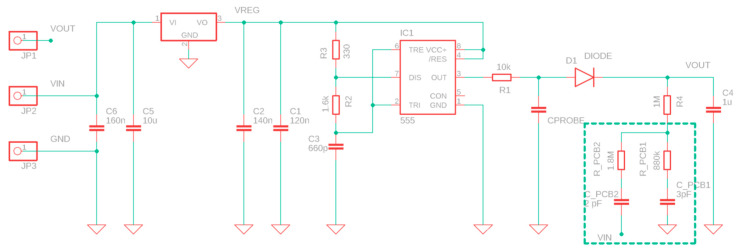
Schematic of the capacitive sensors. Resistance values are directly taken from component labels. Capacitance values have been measured with an impedentiometer at 50 kHz after removal from a particular sample and are affected by ordinary manufacturing errors.

To the best of our knowledge, a data sheet for this type of sensors is only available for version 1.0 [37], manufactured by DFROBOT and advertised with the name SKU:SEN0193. The only significant specifications given in the datasheet are a power supply between 3.3 and 5.5 V, output voltage between 0 and 3 V, and the recommended depth in soil. A detailed analysis of the electrical circuits of the sensor was initially accomplished in order to get acquainted on how the sensor operates (Figure 4).

A low dropout 3.3 V voltage regulator supplies a TL555I CMOS timer whose output signal feeds a low pass filter (10 kΩ resistor and the moisture sensing coplanar capacitor). The main function of this stage is to produce a stationary sawtooth double-exponential waveform whose average value is the same average value of the TL555I output. However, the peak to peak voltage of the waveform depends on the effective dielectric constant of the soil. Then, a peak voltage detector provides the analog output signal that we acquire through the ADC of the microcontroller. During electrical characterization with the sensor probe in air we noted that when an oscilloscope probe is introduced between the 10 kΩ resistor and the diode, then the output voltage is heavily modified, thus indicating that the 14–18 pF and 10 MΩ of the probe significantly modify the circuit behavior. This can be easily understood since the sensor capacitance in air at 1.5 MHz is of the order of 6.5 pF (a value to be confirmed by further measurement and electromagnetic simulations, which are out of the scope of the present paper).

The CPROBE component of Figure 4 corresponds to the section of the sensor immersed in the soil. However, it must be underlined that CPROBE could not be directly associated to the capacitance of Equation (6). In fact, the solder resist dielectric of the sensor separates the soil from the copper electrodes of the sensing capacitor and takes part in the equivalent electric circuit of the sensor. Moreover, this equivalent circuit should also include parasitic capacitances, as shown in the first figure of [35]. 

The peak detector of Figure 4 calculates an analog absolute value of the waveform measured on CPROBE at a constant frequency of about 1.5 MHz. The phase shift caused by the CPROBE equivalent circuit is actually lost in the Vout voltage of this peak detector. Therefore, a real and imaginary part of CPROBE cannot be distinguished using this measurement method. If we add the fact that the equivalent circuit of CPROBE is presently unknown, the only conclusion we could draw is that at constant frequency, the Vout voltage will for sure depend on water content, porosity, and salinity/ionic content of the soil. The chosen “absolute value” measuring method is not capable of discerning among these contributions.

**Figure 5:**
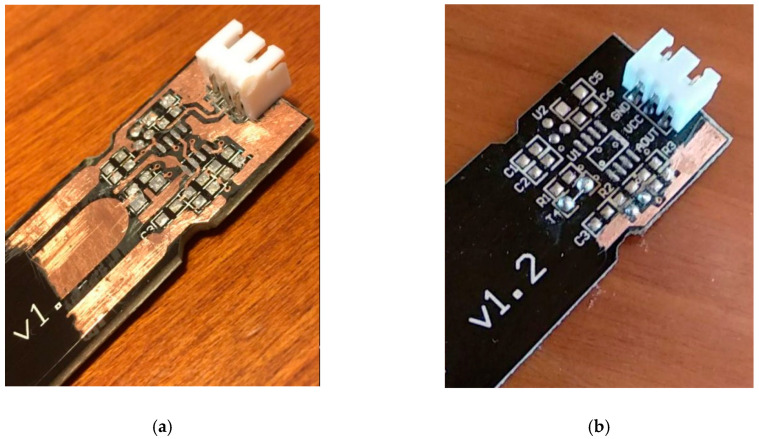
(**a**) Older and (**b**) recent soil moisture sensor v.1.2. The thin metal path to grounded capacitor plate is clearly visible only in (**b**).

Please note that there is no DC path to ground in the peak detector. In fact, the lower node of R4 is connected to a printed circuit board capacitor with parasitic AC interconnects to both GND and Vcc and acts as a voltage divider between Vcc and GND at 1.5 MHz, being the parasitic capacitor impedance of the order of 30 to 60 kΩ, i.e., much smaller than the 1.8 MΩ or 880 kΩ series resistance. This could be interpreted as design fault of the sensor. To confirm this conclusion, a recent (May 2020) batch of v.1.2 sensors shows that R4 is connected to the ground. We removed the passivation of two sensors belonging to the two different batches. Figure 5 clearly shows the missing R4 ground path in the older version of the sensor.

**Figure 6:**
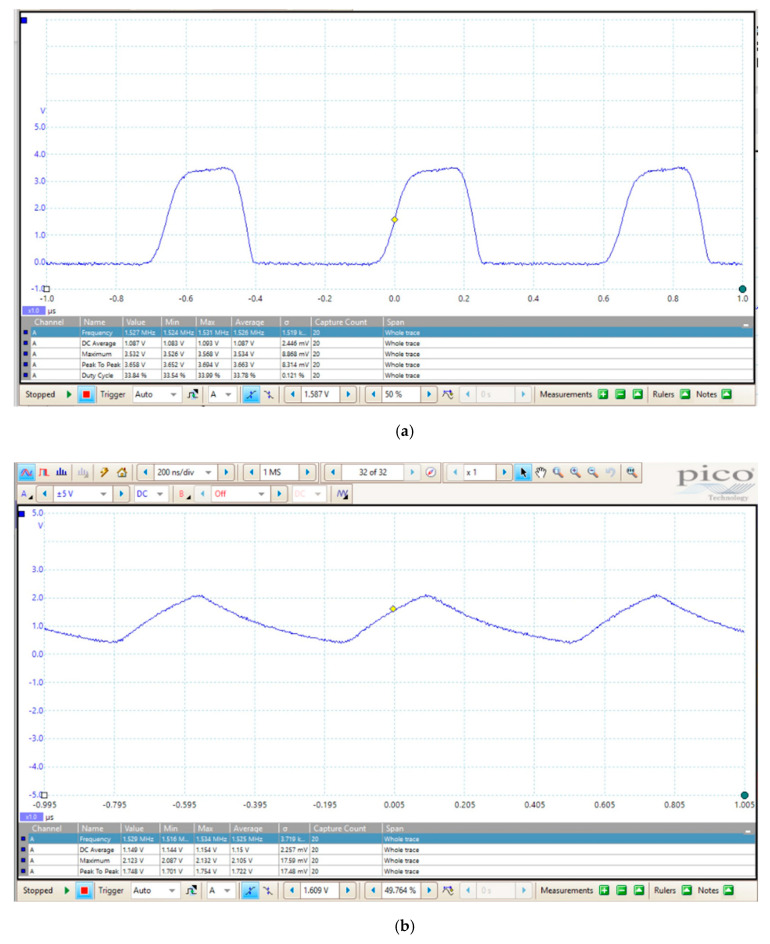
(**a**) TL555I output waveform. The peak voltage exceeds the 3.3 V supply voltage of the TL555I. (**b**) Double exponential waveform on the anode of the diode of Figure 4 measured with a 10 MΩ, 14–18 pF probe connected to the node when the sensor is suspended in air.

The output signal of the TL555I is a trapezoidal waveform running at about a 1.5 MHz (Figure 6a). This trapezoidal profile and the related out-of-specification duty-cycle of about 33% (the duty-cycle of a 555 should always be greater than 50%) are likely caused by the close vicinity of the operating frequency to the physical frequency limit for the TL555I device. On the other hand, it is well known that capacitive soil moisture sensors should operate at a high frequency; the higher the operating frequency, the lower the effect of losses related to the imaginary part of the permittivity. Moreover, the slew rate restraint of the waveform helps in minimizing the electromagnetic interference (EMI), which is possibly generated by the sensor in a non-ISM band and would be beneficial in case of EMI compliance test.

Figure 6b shows the double exponential waveform on the anode of the diode of Figure 4 with a 10 MΩ, 14–18 pF probe connected to the node when the sensor is suspended in air. 

**Figure 7:**
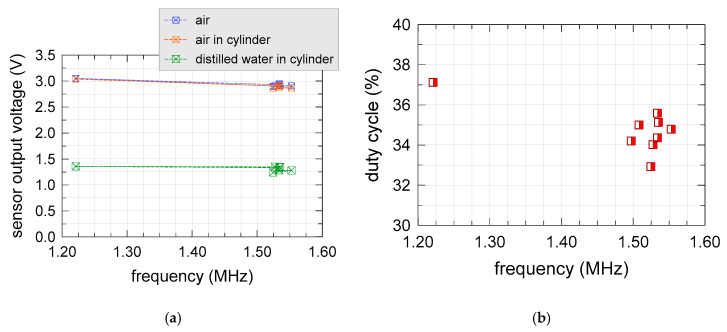
(**a**) Output voltage and (**b**) duty cycle as a function of frequency.

Duty cycle and output voltage of nine different sensors (S1, S2, S5, S6, S7, S9, S10, S13 and S14) were electrically characterized as a function of frequency. Other sensors (S3, S4, S8, S11 and S12) were modified by removing the TL555I and other related components in order to drive them with laboratory waveform generators, or microcontroller boards. The sensor output voltage of the unmodified sensors was measured in three different “standard” conditions: sensor suspended in air, sensor suspended in air within a Delrin^®^ cylinder (with 1.5” inner diameter and 3” height), and sensor suspended in the same Delrin^®^ cylinder filled with distilled water. Results are shown in Figure 7.

Only a single sensor (S1) featured an operating frequency and a duty cycle differed from the other sensors (equal to 1.22 MHz and 37.1%, respectively). The average operating frequency and duty cycle of the other sensors were 1.53 MHz and 34.48%, respectively, with sample standard deviations of 1% and 2.2%, respectively. Electric inspection of the S1 sensor did not show any remarkable difference in R2 and R3 resistance values, compared with the other sensors, indicating that the component most probably responsible for the “freak” behavior was the C3 capacitor or the TL555I itself.

**Figure 8:**
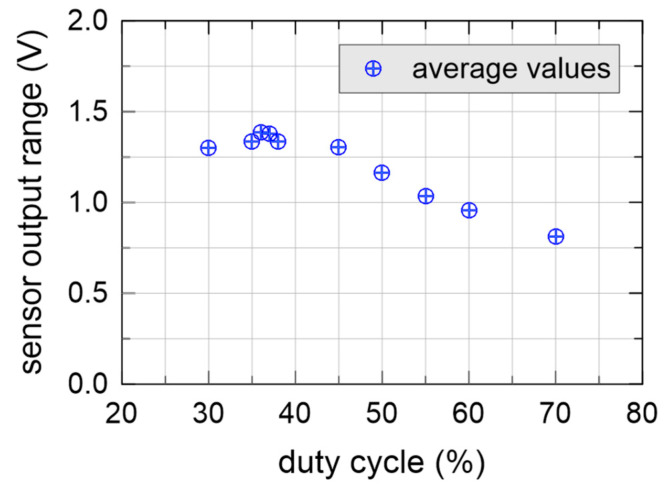
Difference between output voltage in air and in distilled water as a function of duty cycle at 1.5 MHz for a modified sensor driven by laboratory instrumentation.

Figure 8 shows the sensor output range (difference between output voltage in air and in distilled water) as a function of duty cycle at 1.5 MHz for one of the modified sensors, driven by an external waveform generator. A square input waveform was used in this case, with a 3.3 V peak to peak voltage, slightly lower than the peak voltage of Figure 6. The peak of the output range of the sensor lays around duty cycle = 37%, which was slightly higher than the average duty cycle of the non-modified sensors.

For the characterizations of the rest of the paper, we used only two sensors featuring operating frequency and duty cycle shown in Table 1. 

## 4. Experimental Characterization with Silica Sandy Soil

The experimental characterization was planned with the aim to understand how the chosen sensor works in a well-controlled soil environment. To this purpose, we chose to operate with a clean silica sandy soil. In particular, the mineralogical constituents of the soil were SiO_2_ 96% mi., Fe_2_O_3_ 1% max, Al_2_O_3_ 0.5% max, CaO + MgO 1.5% max, and Na_2_O + K_2_O 1.0% max. Commercial distilled water was used for sample preparation.

We chose to prepare our samples using the gravimetric water content (GWC) principle. For volume measurements, we used a 1000 mL graduated cylinder with 20 mL grading divisions.

### 4.1. Sensor Calibration with Constant GWC 

**Figure 9:**
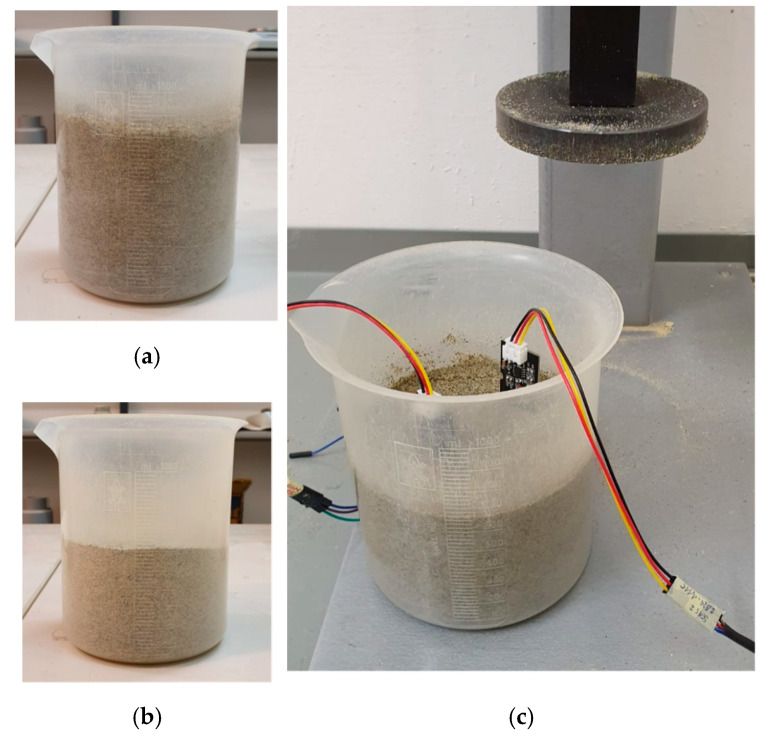
The graduated cylinder: (**a**) no compaction; (**b**) maximum compaction, soil volume is 620 mL; (**c**) soil volume is 680 mL with two sensors driven into.

The first experimental characterization of our work regarded the study of the sensor response with constant GWC of 7.5% using the total sample volume as a parameter. Dry sand was prepared in an oven at 110 °C, then 950 g of material was poured into the graduated cylinder and a gravimetric 7.5% of water was added (Figure 9). The sample was mixed by hand. Then five different samples were prepared in sequence, through dynamic compaction in a graduated cylinder by means of a mortar. For each volume, two capacitive sensors were driven into the soil in two different positions and repeated measurements were acquired.

It should be underlined that the active volume of influence of the coplanar sensor capacitor was much smaller than the total volume of the sample placed in the graduated cylinder.

Sensors were kept sufficiently far apart in order to have no mutual influence between their measurements. After insertion of one sensor in the soil, we determined the minimum distance such that the introduction of the second sensor did not change the reading of the first one.

**Figure 10:**
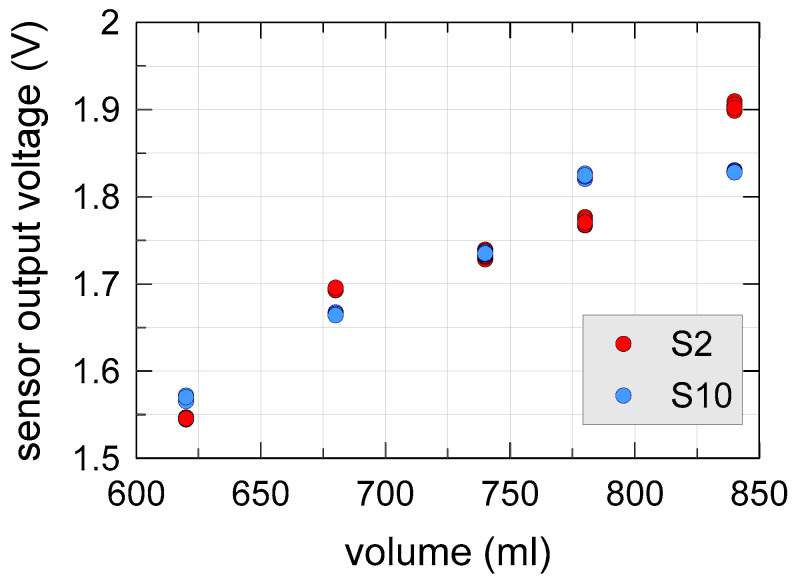
Sensor output voltage as a function of soil volume at constant gravimetric water content (GWC) of 7.5%.

Figure 10 shows the obtained measurement results. Different colors represented the two sensors. Repeated measurements of a single sensor appeared to be very precise, with very low standard deviation (always lower than 3.3 mV). However, measurements of the two sensors differed significantly, even more than 5%. This was most likely due to the non-uniformity of the water content within the soil sample; presumably, the two sensors caught soil regions in the measurement cylinder whereas, due to a different distribution of the pores filled with water, the water content differed too.

The results shown in Figure 10 indicate that sample preparation strongly influences the capacitive sensor measurements. Different levels of soil sample compaction induced significant relative differences of the sensor output voltage. For this reason, we decided to continue the experimental characterization using a constant sample volume.

### 4.2. Sensor Calibration at Constant Soil Volume 

When the capacitive sensor volume of influence did not change during measurements, we supposed we operated at a constant soil volume. This experimental section is devoted to constant volume measurement with GWC as a parameter.

**Figure 11:**
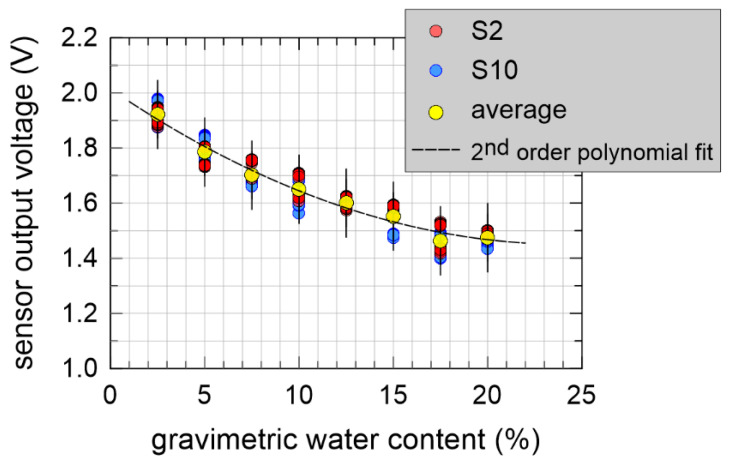
Sensor output voltage as a function of GWC at constant soil volume.

Similarly, to the procedure described in Section 4.1, dry sand was prepared in an oven at 110 °C, then 950 g of silica sand was poured into the graduated cylinder, and eight different weights of distilled water were added, spaced of 2.5%, starting from 2.5% up to 20.0%. The soil-water mixture were obtained by hand. Then, each sample two capacitive sensors were driven into the soil sample in two different positions and repeated measurements were acquired. Figure 11 shows the experimental results. The correlation coefficient of the data was −0.945, relatively far from −1, suggesting a low probability for a linear relationship between the output voltage and the GWC. Error bars represented three times the sample standard deviation, i.e., 0.124 V, calculated with respect to a second order fitting polynomial $V_{out}=A\cdot GWC^{2}+B\cdot GWC+C$. An additional three-parameter exponential fitting $V_{out}=A\cdot e^{GWC/B}+C$ was also attempted and we obtained a very similar threefold sample standard deviation of 0.122.

## 5. Discussion

The experimental results shown in Figure 10 clearly show that that porosity severely affects capacitive soil moisture measurements. If not properly taken into account, this effect could undoubtedly invalidate the results of this type of measurements. Referring to the experimental data of Figure 11, even if the error bars are relatively wide, the constant volume concept helped us obtain a well-defined trend of the output voltage as a function of GWC, with a positive influence on the accuracy of the measurement.

We presently do not know precisely the active volume of influence of the coplanar sensor capacitor. Measurements and electromagnetic simulations are in progress. However, we can assume this volume as a constant even in situ applications, at least for the necessary period of measurement, in the absence of soil volume changes due to any applied actions, including environmental loads. 

Results shown in Section 4.2 demonstrate that, at least for a well-defined type of soil, silica sand in this case, for a constant $\gamma_{dry}$ (see again Equation (2)), coplanar capacitive sensors yielded a reliable relationship between output voltage and gravimetric water content. In addition, for a given soil, viz. for a given value of $\gamma_{dry}$ again, due to Equation (3) our measurements could bring to a corresponding estimate of the volumetric water content. Of course, for different types of soil and for different values of the dry unit weight, a calibration is required.

## 6. Conclusions

The experimental and accurate determination of soil moisture or soil water content is a matter of great importance in different scientific fields. In this paper, a commercial “capacitive” soil moisture sensor typically housed in low-cost distributed nodes for IoT applications was experimentally characterized in order to get acquainted on how the sensor operates. A detailed analysis on the sensor’s electrical circuit was initially carried out. The sensor response with constant GWC using a varying sample volume was investigated. The obtained results indicated that sample preparation strongly influenced the capacitive sensor measurements; different levels of soil sample compaction induced significant relative differences of the sensor output voltage. For this reason, constant sample volume characterizations were carried out and a well-defined trend of the output voltage as a function of GWC was found, as shown in Equation (2). Even if the error bars are relatively wide, the constant volume concept helped us obtain reproducible results, with a positive influence on the accuracy of the measurement. Therefore, at least for a well-defined type of soil at constant volume, the coplanar capacitive sensors yielded a reliable relationship between output voltage and GWC. Although the experimental investigation is still in progress, the results obtained from this study appear to be promising. A possible use of such capacitive sensors for water content measurements in the field will be the object of further research.

## Figures and Tables

**Table 1:** Selected sensors characteristics.

id.	f (MHz)	Duty Cycle
S2	1.53	35.6%
S10	1.51	35%
